# Best Practice Considerations by The American Society of Transplant and Cellular Therapy: Infection Prevention and Management After Chimeric Antigen Receptor T Cell Therapy for Hematological Malignancies

**DOI:** 10.1016/j.jtct.2024.07.018

**Published:** 2024-07-30

**Authors:** Zainab Shahid, Tania Jain, Veronica Dioverti, Martina Pennisi, Lekha Mikkilineni, Swetha Kambhampati Thiruvengadam, Nirali N Shah, Sanjeet Dadwal, Genovefa Papanicolaou, Mehdi Hamadani, Paul A. Carpenter, Gabriela Maron Alfaro, Susan K. Seo, Joshua A. Hill

**Affiliations:** 1 Infectious Diseases Service, Department of Medicine, Memorial Sloan Kettering Cancer Center, New York, New York; 2 Division of Hematological Malignancies and Bone Marrow Transplantation, Sidney Kimmel Comprehensive Cancer Center, Johns Hopkins University, Baltimore, Maryland; 3 Division of Infectious Disease, Department of Medicine, John Hopkins School of Medicine, Baltimore, Maryland; 4 Division of Hematology and Stem Cell Transplantation, Fondazione IRCCS Istituto Nazionale dei Tumori, Milano, Italy; 5 Division of Bone and Marrow Transplant & Cellular Therapies, Stanford School of Medicine, Palo Alto, California; 6 Division of Lymphoma, Department of Hematology & Hematopoietic Cell Transplantation, City of Hope National Medical Center, Duarte, California; 7 Pediatric Oncology Branch, Center for Cancer Research, National Cancer Institute, National Institutes of Health, Bethesda, Maryland; 8 Division of Infectious Disease, Department of Medicine, City of Hope National Medical Center, Duarte, California; 9 Bone Marrow Transplant & Cellular Therapy Program, Medical College of Wisconsin, Milwaukee, Wisconsin; Center for International Blood and Marrow Transplant Research, Milwaukee, Wisconsin; 10 Clinical Research Division, Fred Hutchinson Cancer Center, Seattle, Washington; 11 Department of Infectious Diseases, St. Jude Children’s Research Hospital and Department of Pediatrics, University of Tennessee Health Science Center, Memphis, Tennessee; 12 Vaccine and Infectious Disease Division, Clinical Research Division, Fred Hutchinson Cancer Center, Seattle, Washington

**Keywords:** Infections, CAR T-cell toxicities, Immunocompromised, host, Hematological malignancy

## Abstract

Chimeric antigen receptor (CAR) T-cell therapy is rapidly advancing, offering promising treatments for patients with hematological malignancy. However, associated infectious complications remain a significant concern because of their contribution to patient morbidity and non-relapse mortality. Recent epidemiological insights shed light on risk factors for infections after CAR T-cell therapy. However, the available evidence is predominantly retrospective, highlighting a need for further prospective studies. Institutions are challenged with managing infections after CAR T-cell therapy but variations in the approaches taken underscore the importance of standardizing infection prevention and management protocols across different healthcare settings. Therefore, the Infectious Diseases Special Interest Group of the American Society of Transplantation and Cellular Therapy assembled an expert panel to develop best practice considerations. The aim was to guide healthcare professionals in optimizing infection prevention and management for CAR T-cell therapy recipients and advocates for early consultation of Infectious Diseases during treatment planning phases given the complexities involved. By synthesizing current evidence and expert opinion these best practice considerations provide the basis for understanding infection risk after CAR T-cell therapies and propose risk-mitigating strategies in children, adolescents, and adults. Continued research and collaboration will be essential to refining and effectively implementing these recommendations.

## INTRODUCTION

Chimeric antigen receptor (CAR) T-cell therapies represent a breakthrough in treating hematologic malignancies and are now standard-of-care options for relapsed or refractory (R/R) B cell acute lymphoblastic leukemia (B-ALL); B cell non-Hodgkin-lymphomas (NHL) including diffuse large B cell lymphoma (DLBCL), follicular lymphoma (FL), mantle cell lymphoma (MCL); and multiple myeloma (MM) [1–5]. While cytokine release syndrome (CRS) and immune effector cell associated neurotoxicity syndrome (ICANS) are well-known immediate toxicities, infections are increasingly being identified, particularly in the first 30 d, and can add to nonrelapse mortality following CAR T-cell therapy [6–9]. These best practice considerations aim to explain the basis for understanding infection risk after CAR T-cell therapies and propose risk-mitigating strategies in children, adolescents, and adults.

The American Society for Transplantation and Cellular Therapy (ASTCT) Committee on Practice Guidelines (CoPG) and ASTCT Transplant Infectious Disease Special Interest Group (TID-SIG) collectively developed an expert opinion panel in the field, to identify key challenges in the management of infections after CAR T-cell therapy. Ten key clinical practice questions were developed, and best practice recommendations were formulated. The responses were formulated based on published literature and mutually agreed upon expert opinions of the panel. Mutual agreement on recommendations was reached through a series of email correspondence.

### FAQ1: What is the Frequency of Infections Among CAR T-Cell Therapy Recipients?

A recent large meta-analysis reported 33.8%; 16.2% were severe (grade ≥3), and pooled attributable mortality was 1.8% in adults (I^2^ = 43.44%) [10]. A similar 22% to 40% frequency has been reported in children, adolescents, and young adults (CAYA) [2,11–15]. Types of infections may vary over time, depending on the tempo of immune recovery, which may also differ according to the underlying disease and type of CAR T-cell product:

#### CD19 CAR T-Cells

- Infection density is higher before d 30 versus after d 30 [11–17]; bacterial infections tend to be more frequent during the first 30 d.
- After d 30, respiratory viral infections appear most common [12,13,16,18,19].
- Fungal infections are <6% [12] while viral reactivations (Herpes Simplex Virus (HSV), Varicella Zoster Virus (VZV), Human Herpes Virus −6 (HHV-6), Cytomegalovirus (CMV), adenovirus) are rare, but occur before and after d 30, (acyclovir prophylaxis is common practice). [20–23]

#### BCMA CAR T-Cells

- Infection rates reported in myeloma clinical trials were high at 58% to 69% (20% to 48% ≥grade 3) but occurred later (median 46–60 d [15,19]) than those reported after anti-CD19 CAR T-cell therapy; infection density is highest between d 30 and 100 and declines beyond d 100 [4,24].
- Respiratory viral infections are prevalent before and after d 30 [25,26]
- Fungal infections are uncommon (<5%), and virus reactivations (Epstein Barr Virus (EBV), CMV, hepatitis B virus (HBV), VZV have rarely been observed [22,27].

### FAQ 2: What are the Risk Factors for Infection After CAR T-Cell Therapy?

**Figure 1.**
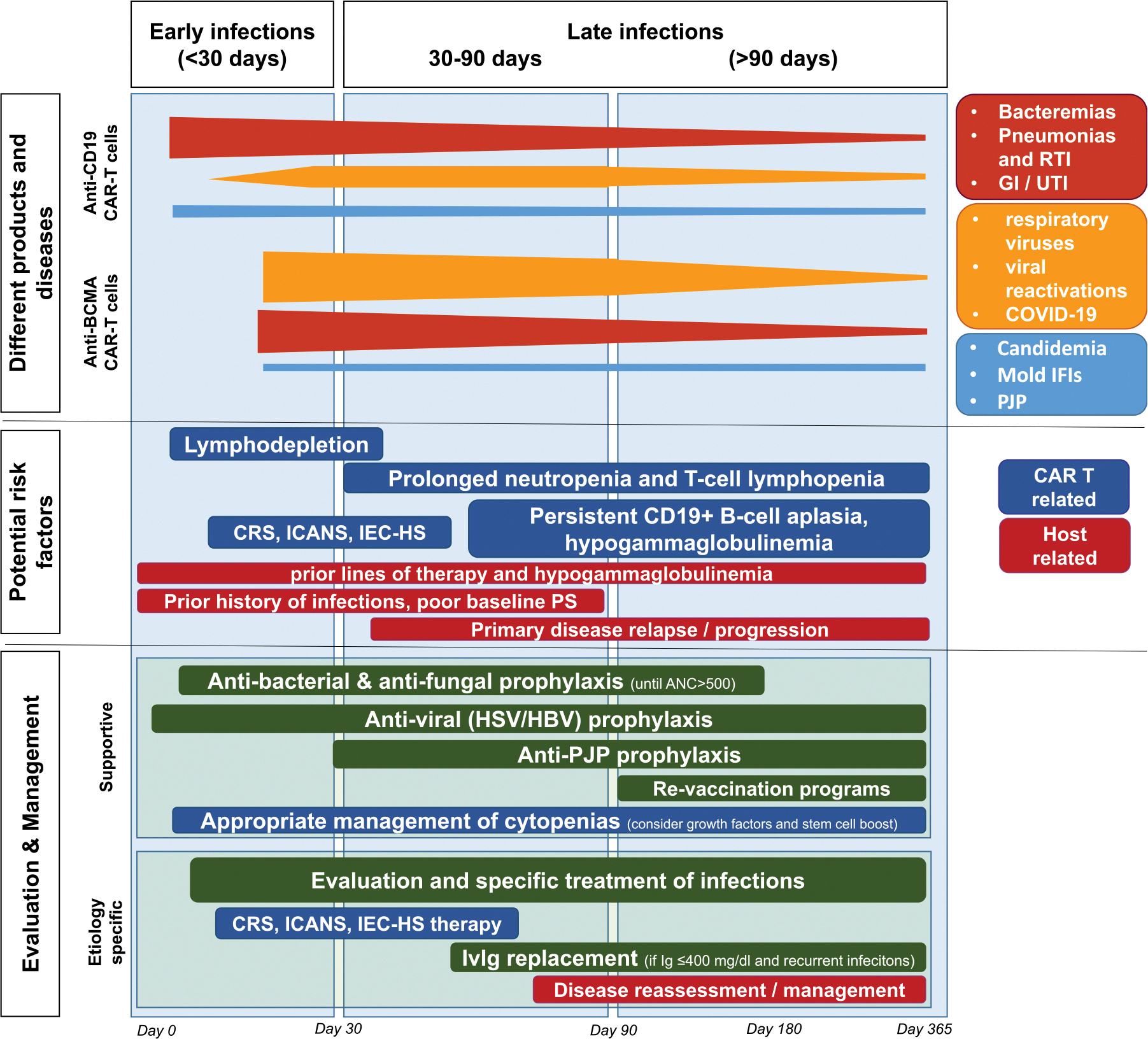
Outline of infections occurrence, possible etiologies and management. The majority of infections tend to occur during the first 30 to 90 d after CAR T cells infusion, then the occurrence of infections decreases over time. Early events (<30 d) are largely related to the initial toxicity of lymphodepleting chemotherapy and are dominated by bacterial infections, followed by viral and then fungal infections. Infections occurring after d 30 are mainly caused by CAR T cells-associated prolonged neutropenia and T cells lymphopenia, and by B-cells aplasia and hypogammaglobulinemia. Late infections (≥30 d) are largely represented by viral infections immediately followed by bacterial infections, then by fungal infections. In multiple myeloma patients treated with anti-BCMA CAR T cells, infections tend to occur more frequently but later than in patients treated with anti-CD19 CAR T cells. Majority of events occur by d 100 after infusion and are represented largely by respiratory tract viral and bacterial infections. CRS, Cytokine Release Syndrome; GI, Gastrointestinal Infections; HBV, Hepatitis B Virus; HSV, herpes simplex virus; ICANS, immune effector cell-associated neurotoxicity syndrome; IEC-HS, immune effector cell-associated hemophagocytic lymphohistiocytosis-like syndrome; IFI, invasive fungal infections; IvIg, intravenous immunoglobulin; PJP, pneumocystis jirovecii pneumonia; PS, performance status; RTI, respiratory tract infections; UTI, urinary tract infections. (This figure is available in color online at www.astctjournal.org.)

Although results of the informative retrospective observational studies have sometimes been conflicting, risk factors have been categorized as pre-existing patient-related or CAR T-cell related (Table 1). Several risk factors might coexist, so determining the dominant risk attribution during infection assessments, while often challenging, is important because it aids in categorizing individuals as having heightened susceptibility to infections and can inform the choice of prophylactic and preemptive clinical practice strategies. Figure 1 outlines the epidemiology of infections, their risk factors, and potential management strategies.

### FAQ3: What Infection Screening is Recommended Before CAR T Therapy?

All patients should undergo a detailed history (including vaccinations) and physical examination before lymphodepleting chemotherapy and CAR T-cell infusion. Any new symptoms and/or physical findings should be thoroughly investigated with Infectious Disease (ID) consultation as appropriate. If an infection is identified, a multidisciplinary discussion regarding the risks of infection progression with lymphodepleting therapy versus the risks of progression of underlying malignancy is recommended to decide on the best approach.

#### Routine Screening Prior to CAR T-Cell Therapy

- Laboratory screening should include HIV antibodies (preferably fourth-generation antigen/antibody combination), hepatitis B virus surface antibody (anti-HBs) and antigen (HBsAg), HBV core Ab (anti-HBc), hepatitis C virus antibody (anti-HCV) with reflex nucleic acid testing if positive; Treponema pallidum testing based on risk factors (screening per institutional guidelines with treponemal or nontreponemal testing).
- Patients with positive HBsAg should get baseline HBV DNA.
- Patients with respiratory symptoms should be tested for respiratory viruses via multiplex viral PCR, including SARS-CoV-2, and undergo lung imaging as indicated. The utility of screening asymptomatic adult patients is unclear [28] and the practice is evolving in CAYA, with many centers only testing symptomatic patients. Patients with severe symptomatic lower respiratory tract infections, especially SARS-CoV-2, should be delayed until clinically improved; ASTCT guidelines recommend a delay of 14–20 d for those with symptomatic infection [29]. Patients with asymptomatic or mild respiratory viral infections might proceed if delays in CAR T-cell therapy are deemed too high risk for the progression of the underlying malignancy [30,31]; multidisciplinary discussion including Infectious Disease expertise is recommended.

#### Patient-Specific Screening if Known Risk Factors Present

- Mycobacterium tuberculosis testing: a negative skin or blood TB test in a patient from an endemic area should be carefully interpreted in the setting of lymphopenia and/or extensive prior lymphodepleting chemotherapy.
- Strongyloides stercoralis antibody screening (versus empiric treatment with ivermectin) since potential high-dose corticosteroids for CRS and/or ICANS treatment increase the risk for hyperinfection syndrome.
- Toxoplasma gondii serologies for patients with a history of exposure.
- Check serologies for HSV1, HSV2, and VZV only if prophylaxis against these viruses is not universally practiced. Also, consider testing for CMV and Human T-cell Lymphotropic virus type (HTLV-1) serostatus.

Table 2 summarizes infection screening recommendations prior to CAR T-cell therapy.

### FAQ 4: What Antimicrobial Prophylaxis is Recommended After CAR T-Cell Therapy?

Because clinical trials have not yet addressed this topic, there are no relevant clinical standards. Infection risk is highest for the first 30 d after CAR T-cell infusion, likely related to neutropenia and further immunosuppression needed to treat CRS and/or ICANS [16]. Most centers extrapolate prophylaxis recommendations from hematopoietic cell transplantation (HCT) and expert opinion, although there is variability in agent, timing, and duration. The overall strategy considers prophylaxis against bacteria, *Pneumocystis jirovecii,* certain viruses, and fungi, and provides recommended stopping criteria (Table 3).

#### Antibacterial

- Routine prophylaxis with fluoroquinolones (alternatively 3rd generation oral cephalosporins) remains controversial [32–34]. Some centers that perform inpatient CAR T-cell therapy might alternatively consider careful monitoring and initiation of intravenous antibiotics in those who become febrile while neutropenic; none of the pediatric studies utilized antimicrobial prophylaxis [11–15].
- In the outpatient setting, fluoroquinolone prophylaxis can be considered during the expected period of neutropenia through count recovery (ANC >0.5 × 10^9^/L on 2 consecutive days) [35].
- The 11% to 17% of CAR T-cell recipients who have persistent cytopenias beyond d 30 (sometimes ≥3 mo without evidence of disease relapse or bone marrow dysplasia [2,18,20,32,36]), may require longer prophylaxis [37].
- Antiviral
- HSV and VZV should be prevented with acyclovir or valacyclovir from the start of lymphodepletion therapy until at least 6 mo postinfusion. Pediatric populations are usually given shorter (often 3 mo, range 1 to 6 in clinical studies) duration [14]. HSV/VZV infection rate is low (FAQ1) due to acyclovir prophylaxis. The incidence of herpes virus infections, particularly VZV (median onset 79 d, range 49 to 723 d), was found to be as high as 50% if antiviral prophylaxis was not instituted [38].
- Currently, there is insufficient data to support the recommendation of CMV prophylaxis for CMV seropositive patients. In a patient with a rising CMV viral load treatment threshold is unknown without CMV disease [23], ID consultation should be requested for further management.
- Although routine monitoring for CMV, EBV, and HHV-6, is not recommended but targeted monitoring can be considered in special populations [39,40] (see FAQ6).
- Patients who test positive for hepatitis B surface antigen (HBsAg) should receive entecavir 0.5 mg once daily beginning at lymphodepletion chemotherapy until at least ≥12 mo post-CAR T; early discontinuation can lead to fatal reactivation [41–44].
- Chronic HBV carriers (HBsAg negative, anti-HBc positive) had a 40.8% 2-year post-HCT cumulative risk for reactivation [45]. By extrapolation, they should either receive prophylaxis in the CAR T-cell therapy context [46,47], or be monitored with monthly HBV DNA and liver enzymes to detect early reactivation and, if so, initiate treatment [41].
- Patients with chronic hepatitis C virus (HCV) should be referred to hepatology for further evaluation of candidacy for direct-acting antiviral (DAA) therapy [46].
- Given limited data regarding outcomes of CAR T-cell therapy in patients living with human immunodeficiency virus (HIV), they should be managed in consultation with an HIV specialist to control viral load before starting lymphodepletion [48]. Patients should continue antiretroviral therapy with minimal interruptions.

#### Antifungal

- For centers that practice universal antifungal prophylaxis, fluconazole can begin with lymphodepletion to cover the neutropenic period until neutrophil recovery.
- Because rates of invasive fungal infections (IFI) have varied from 0% to 15% [11,16–18,49,50, 51,52], selected patients can be considered for mold-active antifungal prophylaxis, including children [11,13,14], based on risk factors that include persistent neutropenia, severe CRS, recipients of high-dose corticosteroids, and centers where Aspergillosis rates are ≥6% [16,33,51,53, 54]. Bruton’s tyrosine kinase inhibitors given prior to CAR T cell therapy, may also increase the risk of IFI after therapy [55].
- Prophylaxis against Pneumocystis jirovecii (PJP) with trimethoprim-sulfamethoxazole (TMP/SMX) is recommended to start post-CAR T cell infusion until at least 6 mo. This is based on a study that showed 2 of 40 patients developed PJP pneumonia <6 mo post-CAR T cell therapy when prophylaxis was routinely discontinued at 3 mo; all cases were severe and required mechanical ventilation [20]. Whether total CD4+ T cell count should guide discontinuation of prophylaxis is unknown, but some centers have adopted a threshold of <200 cell/mm^3^ to guide duration [56]. Less marrow suppressive alternative medications are considered for patients with prolonged cytopenias (Table 3).

### FAQ 5: How to Approach Patients With Recent Infections Before CAR T-Cell Therapy?

The lack of prospective or large-scale retrospective study data to inform risk assessment for proceeding to lymphodepletion and then CAR T-cell infusion with active infection means that medical decision-making is based mainly on general principles and extrapolation from other data. Infections within 100 d of lymphodepletion have been associated with increased infectious risk within 30 d of CAR T infusion. [11,57,58]. A study of adults with B-cell malignancies who received anti-CD19 CAR T-cells reported that baseline gut microbiome composition was correlated with clinical response and that prior treatment with broad-spectrum antibiotics was associated with worse survival and increased neurotoxicity [59]. Taking these important observations together, the panel recommendations are to:

- Involve ID specialists during eligibility screening to create a plan that balances the urgency for proceeding to CAR T-cell therapy versus post-CAR T-cell infusion morbidities, including infectious risks, in collaboration with the oncologist.
- Delay therapy until the infection is controlled, if possible.
- Tailor treatment or appropriate prophylaxis if proceeding to CAR T-cell therapy.
- Develop a surveillance plan for infections the patient is at high risk for after lymphodepletion.

### FAQ 6. What Infections Should be Monitored, and How Long After CAR T-Cell Infusion?

Patients should have a history and physical at each follow-up visit to ensure no active infections are present. Patients with a high risk for fungal infections should be monitored clinically and potentially started on anti-mold prophylaxis if appropriate.

Data regarding the epidemiology and clinical significance of viral infections such as CMV, HHV6, EBV, and adenovirus in patients receiving CAR T-cell therapy are limited. Prospective CMV surveillance has shown a reactivation upwards of 25%, with increased risk among BCMA CAR T recipients and >3 d of steroid use [23,60,61]. Others have reported both CMV and HHV-6 reactivation as well as disease [32,62–65,61, 66,67]. Management of CRS and ICANS often requires multiple days of corticosteroid and immunosuppressive therapy, increasing the risk of reactivation and infection with these viruses [21]. Immune effector cell-associated Hemophagocytic Lymphohistiocytosis-like Syndrome (IEC-HS), another toxicity of CAR T therapy, requires significant immunosuppression, leading to increased infection risk with these viruses. Without end-organ disease, the optimal approach to managing CMV reactivation and other viruses remains uncertain because treatment thresholds are unknown.

Weekly CMV surveillance can be considered in the first 2 to 6 wk post-CAR T infusion in patients with a risk high risk of CMV reactivation such as those with a prior history of CMV reactivation, >3 d of corticosteroid use, receipt of BCMA CAR T products, and more lines of chemotherapy prior to CAR T cell therapy [60]. Another example would be heavily treated pediatric ALL patients or those receiving prolonged immunosuppressive therapies for CAR T cell toxicities. The decision to treat depends on the clinical situation and institutional practices.

The panel does not recommend routine EBV, HHV-6, and adenovirus PCR surveillance. Certain investigational protocols may require escalated lymphodepletion or T cell depleting strategies, particularly in allogeneic CARs, and can be high-risk settings for viral reactivation; additional screening may be warranted in such settings.

#### Summary of Recommendations

- Patients should have CMV serology at baseline.
- In patients at high risk for viral reactivation or infection or those with sequelae of infectious or neurological symptoms that are unexplained by alternative diagnoses, viral PCRs should be checked.

### FAQ 7: What is the Management of Fever Post-CAR T Infection?

Fevers occur in 24% to 94% of patients after CAR T-cell infusions [64] with CRS being a common cause that can be refractory or recurrent [68,69]. Other CRS symptoms include malaise, fatigue, headache, nausea, hypotension, and end-organ dysfunction [70]. CRS usually manifests within the first 2 wk of CAR T-cell infusion with a median time to onset of 2 to 3 d [2,71,72]. CRS treatment with interleukin-6 (IL-6) and/or steroids may alter or mask the clinical presentation of infection, especially in the setting of concomitant neutropenia. Cytokine profiling to differentiate between CRS and severe infection is still evolving and is not utilized routinely to make clinical decisions [73].

Fever within 30 d after CAR T-cell infusion, irrespective of neutrophil count, should prompt infectious workup. ID consultation should be considered early [74–76] and workup should include blood cultures, urinalysis, urine culture, radiographic imaging, and additional testing (eg, *Clostridioides difficile* stool test, respiratory pathogen panel from the upper respiratory tract) as indicated by the clinical scenario [16,77]. Pulmonary toxicities associated with CAR T-cell therapy can mimic infections, and bronchoalveolar lavage might help with diagnosis [78].

**Figure 2.**
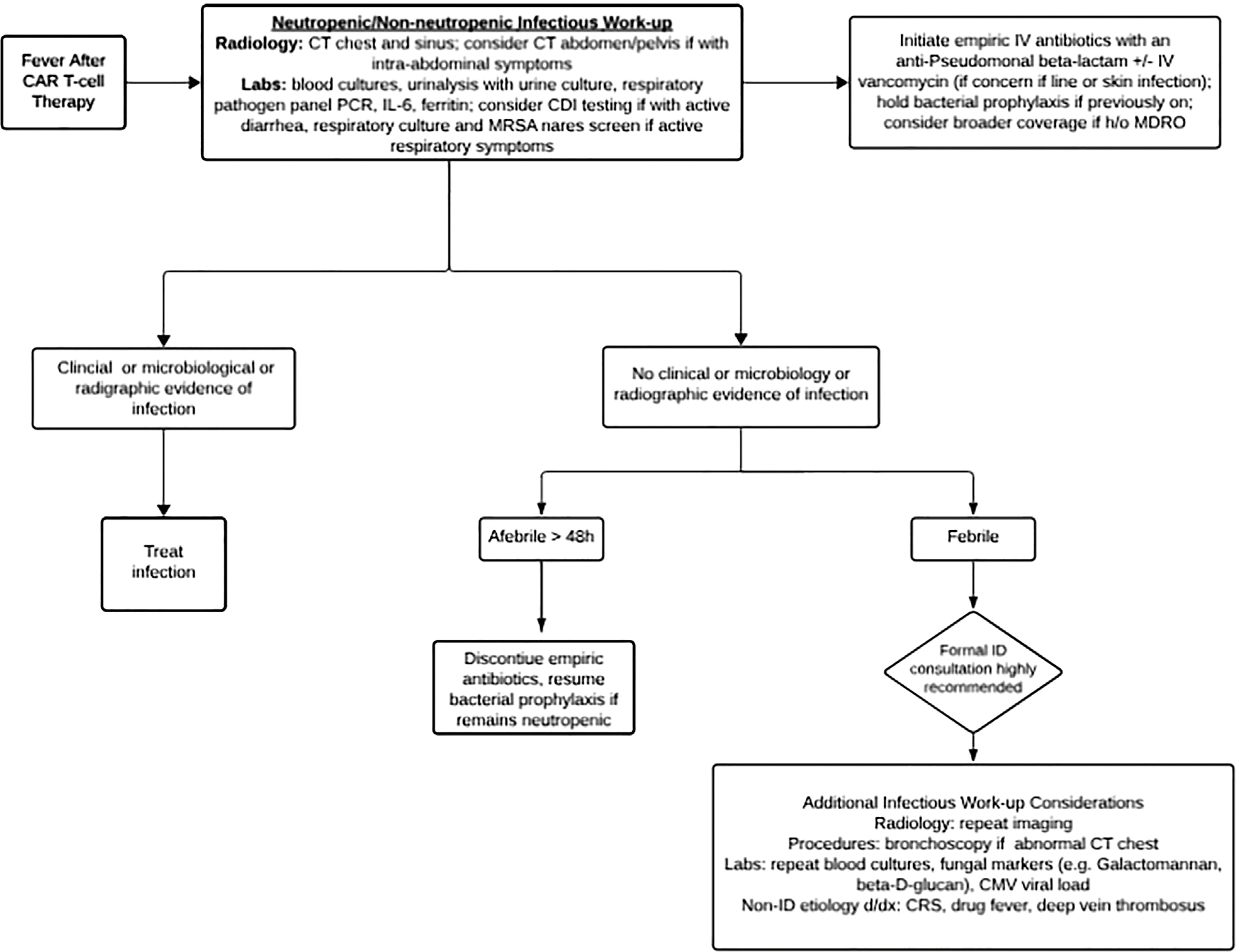
Flow diagram for management of fever after CAR T-cell therapy.

Given the predominance of bacterial infections early after CAR T-cell therapy, there should be a low threshold for rapidly initiating broad-spectrum, bactericidal antibiotics per institutional guidelines for the management of fever and neutropenia. [79,80]. Antimicrobial therapy can be modified if there is clinical and/or microbiological evidence for infection. If respiratory viral infections are identified as the source of fever, their management should follow the guidelines for patients with hematological malignancies and TCT [29,81,82]. An antibiotic de-escalation strategy, followed by close monitoring, may be safe and can be considered if there is no clinical suspicion of infection and the fever resolves [83]. Figure 2 summarizes fever management post CAR T cell therapy.

#### Summary of Recommendations

- Because CRS treatment can mask fever, clinical suspicion for infection should be high, and early ID consultation should be considered.
- Fevers after CAR T-cell infusion should warrant prompt infectious workup and initiation of broad-spectrum antibiotics.

### FAQ8: What is the Management of Hypogammaglobulinemia?

CAR T-cell therapies targeting the B-lineage surface antigen CD19 and the plasma cell surface antigen BCMA are associated with hypogammaglobulinemia which may increase the risk of infection, especially of encapsulated bacteria [84]. The incidence of hypogammaglobulinemia after CD19-directed CAR T-cell therapy has varied greatly, 23% to 81%, with up to 44% of patients having hypogammaglobulinemia for ≥18 mo [84–87]. BCMA CAR T-cell therapy is associated with hypogammaglobulinemia in up to 69% of patients between d 60 to 90, and 42% to 47% showing persistence >1 yr [4,88–90]. Hypogammaglobulinemia may predate CAR T-cell therapy due to prior B-cell-directed therapy (23% to 44%) [16]*.* While CD19 CAR T-cell therapy can lead to universal B-cell aplasia and secondary hypogammaglobulinemia, BCMA CAR T therapy may have a greater impact on preexisting pathogen-specific immunity while preserving naïve and memory B-cell populations. [91]

### Intravenous Immunoglobulin G (IVIG) Supplementation Recommendations Post-CAR T

Evidence from randomized clinical trials to support empiric IVIG supplementation after CAR T-cell therapy is lacking. Limited data plus study heterogeneity hinders comparisons due to variations in CAR T-cell products, patient demographics, and IVIG practice guidelines variation. While some institutions administer supplemental IVIG for IgG ≤ 400 mg/dL others require patients to also have a history of recurrent or severe infections. [84] Routine supplementation has been recommended for children, adolescents, and young adults whenever IgG ≤400 mg/dL [12–15,92].

#### Summary of Recommendations

- For B cell malignancies, we recommend measuring serum immunoglobulin G (IgG) before and in the first 3 mo after CAR T-cell therapy. IVIG administration can be considered in the weeks before LD initiation, and as prophylaxis after infusion if there is a prior history of recurrent infections and persistent hypogammaglobulinemia [84,93].
- In aligning our guidelines for IVIG supplementation after CAR T-cell therapy with the ASBMT/EBMT “*Choosing Wisely*” guidance for IVIG supplementation after HCT, [94] we recommend considering IVIG in patients with IgG ≤ 400 who are experiencing severe or recurrent bacterial infections, particularly involving the sinopulmonary tract. [84]
- Beyond the first 3 mo, patients with IgG ≤ 400 mg/dL who are not experiencing infections may be trialed off IVIG with close monitoring. IVIG supplementation may be considered for patients with IgG 400mg/dL-600mg/dL who are experiencing recurrent or severe bacterial infections [84]. IVIG can also be considered when IgG < 200 mg/dL, particularly when associated with extremely low IgA levels, irrespective of the time from CAR T-cell infusion and the incidence and severity of infections [86].
- Patients with IgG > 600 mg/dL with recurrent or serious infections should undergo additional immunologic evaluation, and if specific IgG subclass levels or immunization titers are low or nonprotective, IgG replacement or vaccination can be considered [84].
- IgG supplementation should be given with 400 to 500 mg/kg IVIG [91] every 3 to 4 wk [95,96] with a goal IgG trough > 600 mg/dL in adults [95].
- If a patient continues to have recurrent infections despite monthly supplementation, the dose or frequency can be increased, or subcutaneous (SC) administration with a specific SCIg formulation can be considered [91,92].
- There is currently no evidence to support the safety of stopping immune globulin in children. Supplementation should be given with 250 to 500 mg/kg IVIG every 4 wk. In children with prolonged B-cell aplasia, SCIg replacement may be considered, with a goal to maintain IgG > 1000 mg/dL [92].

### FAQ9: What are the Recommendations for Revaccination After CAR T Therapy?

Current recommendations are largely extrapolated from other immunocompromised and HCT populations, although several studies have been reported using SARS-CoV-2 mRNA vaccines [97–99]. More research is needed to identify factors associated with vaccine immunogenicity in CAR T-cell therapy recipients and to optimize revaccination schedules. Two major points to consider:

- Although vaccine responses may be lower in CAR T recipients compared to immunocompetent individuals, humoral immunity may be elicited, and cellular immune responses may be more robust, to help prevent infection severity [97,100,101].
- It is unclear to what degree CAR T-cell therapy impacts previously established humoral immunity to vaccine-preventable infections, although this appears to be relatively well preserved in CD19 CAR T-cell recipients and less so after BCMA CAR T cell therapy [102].

#### FAQ9.1: Which vaccines should be administered before CAR T-cell therapy?

- Because diminished immunologic responses can be anticipated immediately after CAR T-cell therapy, there are no recommendations to administer vaccinations prior to CAR T-cell therapy other than the seasonal inactivated influenza vaccine (IIV) and vaccines directed against SARS-CoV-2 [103].

#### FAQ9.2: Which vaccines should be administered after CAR T-cell therapy?

- At a minimum, we recommend CAR T-cell recipients should be (re)vaccinated for *S. pneumoniae*, hepatitis A, hepatitis B (if lacking seroprotection), *C. tetani, C. diphtheriae*, and *B. pertussis*. VZV seropositive adult recipients or those with a history of chickenpox or shingles may be administered the recombinant adjuvanted zoster vaccine. SARS-CoV-2 vaccination is discussed below. Table 4 shows the vaccination schema in adult patients who have received CAR T-cell therapy [91]; pediatric guidance is not provided due to the lack of data.

#### FAQ9.2: When should vaccine administration begin after CAR T-cell therapy?

- If CAR T-cell is the final therapy and the patient is in remission, inactivated vaccines may be considered ≥6 mo after CAR T therapy and live and non-live adjuvant vaccines may be considered ≥ 1 yr after CAR T-cell therapy is no longer considered immunocompromised [91]. For endemic or seasonal infections (e.g., influenza and SARS-CoV-2), vaccinations should be started around 3 mo after CAR T-cell therapy.

#### FAQ9.3: How to approach CAR T-cell recipients who were prior HCT recipients who did not complete post-HCT vaccinations?

- For patients without a history of HCT or who completed the entire series of post-HCT vaccines, no additional vaccinations are needed if the patient developed *seroprotective titers* to the first post-CAR T-cell therapy vaccine series with pneumococcal conjugate vaccine, DTaP, Hepatitis A, or Hepatitis B vaccines.
- Patients who did not complete the entire series of post-HCT vaccines but had a positive response to the first post-CAR T-cell therapy vaccines should complete the rest of the post-HCT series as indicated. Patients who did not respond to the first post-CAR T-cell vaccines should defer additional vaccination until improved immune reconstitution is documented [91].

#### FAQ9.4: How to consider SARS-CoV-2 vaccination after CAR T-cell therapy?

- Although only one-third of patients develop a high level of antibodies to SARS-CoV-2 vaccination, T-cell responses appear to be more frequent, and delaying vaccination may not improve immunogenicity [97]. Thus, the currently available mRNA SARS-CoV-2 vaccine series should be started as early as 3 mo post-CAR T-cell therapy [98]. Patients should be fully revaccinated with a primary series (currently 3 doses for mRNA vaccines) and a booster [104]. Additional boosters might be recommended; the vaccination schedule with vaccines can be found on the Centers for Disease Control website [105].

#### FAQ9.5: How to consider RSV vaccination after CAR T-cell therapy?

- Two Respiratory Syncytial Virus (RSV) vaccines were recently approved for individuals ≥ over 60, but immunogenicity and efficacy are unknown in CAR T-cell recipients [106].

### FAQ10: What are Future Directions for Preventing and Treating Infectious Complications After CAR T Therapies?

Evidence to inform these best practice considerations is largely from retrospective, uncontrolled studies, and long-term follow-up after CAR T-cell therapy is lacking. Thus, the associated lifetime infection risk is not fully understood. Immunosuppression after CAR T-cell therapy is influenced by multiple factors, and the pace of immune reconstitution varies. Areas for future research and ongoing CAR T cell therapy knowledge gaps include (see also Table 5):

- Lack of prospective studies about infection surveillance or the type and duration of infection prophylaxis.
- Efficacy and optimal use of IgG supplementation.
- Determining the optimal timing and schedule for vaccinations.
- Microbiota consideration and optimal use of antibiotics.
- Enhanced diagnostics, including an omics-based approach, are needed to better understand immune phenomena and infection risk.
- Integration of Artificial Intelligence (AI) tools to enhance infection-related patient care outcomes.

By attending to these forthcoming avenues and informational voids, the field of CAR T-cell therapy can further refine its protocols and enhance patient outcomes.

## Figures and Tables

**Table 1:** Risk Factors Associated with Infections Risk After CAR T Therapy

Host Related Risk Factors	References
Prior lines of pre-CAR T therapy	[10,15,19,48]
Prior history of transplantation	[11,12]
Underlying malignancy, B-ALL and MM	[10,15,107]
CAR-Hematotox Score >2, lymphopenia	[11,70,103,108,109]
Prior history of infection within 100 d	[10,55,48]
Performance status at baseline	[25,55]
Baseline Hypogammaglobulinemia*	[11,55]
Older age^†^	[14]
**CAR T Related Risk Factors**	
High CAR T dose and intensity of lymphodepletion	[15,11]
Presence and severity of CRS	[11,15,16]
Immune effector cell-associated hematotoxicity^‡^ (ICHAT)	[25,105,110]
Corticosteroid use	[10,19,26,55,82,104,111]
Hypogammaglobulinemia post CAR T	[24,25]
IEC-HS^§^	[18]

* Threshold “low” IgG definition can vary.

† Calculated as adults vs children

‡ Includes thrombocytopenia, anemia, and neutropenia.

§ Immune effector cell- associated hemophagocytic-lymphohistiocytosis-like syndrome

**Table 2:** Recommended Infection Screening

Routine:

○ HIV antibody (reflex nucleic acid testing if positive)
○ HBsAg, anti-HBc and anti-HBs*
○ HCV IgG (reflex nucleic acid testing if positive)
○ *Treponema pallidum*
○ CMV IgG
○ HTLV-1 IgG

*If respiratory symptoms present:*

○ Multiplex nasal swab PCR for respiratory viruses

*If antiviral prophylaxis not universally practiced:*

○ HSV-1 and 2 IgG
○ VZV IgG

Based on risk factors:

○ Toxoplasma gondii IgG
○ Mycobacterium tuberculosis blood test
○ Strongyloides stercolaris^†^ IgG
○ Endemic mycosis serologies^‡^

* If HBsAg is positive, obtain baseline HBV DNA PCR.

† Based on the geographic risk.

‡ Histoplasma and coccidiosis serology.

**Table 3:** Recommended Antimicrobial Prophylaxis Before and After CAR T Therapy

	Agent	Dose Regimen	Duration
Antibacterials*	Fluoroquinolones		At lymphodepletion therapy until 2 consecutive days of ANC >0.5 × 10^9^/L
	- Levofloxacin	500–750 mg PO QD	
	- Moxifloxacin	400 mg PO QD	
Antivirals			
- HSV/VZV	Acyclovir Valacyclovir	400 mg PO BID 500 mg PO BID	At lymphodepletion therapy until ≥6 m post-CAR T
- HBV	Entecavir	0.5 mg PO QD	At lymphodepletion therapy until ≥12 m post-CAR T
Antifungals^†^			
	Fluconazole^‡^ Echinocandins^§^	200 mg PO QD	Start with lymphodepletion therapy until 2 consecutive days of ANC >0.5 × 10^9^/L
*Pneumocysitis jirovecii*	TMP/SMX Dapsone Atovaquone Pentamidine	1 SS tablet PO QD or 1 DS PO TIW 100 mg PO QD 1500 mg PO QD inhaled or IV monthly	Start with ANC recovery (at least by d 30 post-CAR T) therapy until ≥6 m post-CAR T

Abbreviations: ANC, absolute neutrophil count; CTI, cell therapy infusion; HBV, hepatitis B virus; HSV, herpes simplex virus; BID, twice daily; DS, double strength; QD, once daily; TIW, three times per week; TMP/SMX, trimethoprim/sulfamethoxazole.

* Alternatively, consider close monitoring instead of antibacterial prophylaxis; oral cephalosporins if fluoroquinolones are contraindicated. In children, adolescents and young adults, antibacterial prophylaxis is not universally recommended.

† Consider mold active azoles if prior allo-HCT, prolonged neutropenia, corticosteroid therapy for CAR T-cell-related toxicities, and/or recent history of mold infection. Dosing will depend on echinocandin of choice.

‡ Extended fluconazole prophylaxis is recommended by some experts for those living in coccidioides endemic areas and/or with positive coccidiodes serology in high-risk setting, based on extrapolation from an allo-HCT study which showed high mortality rate [112]

§ If fluconazole is contraindicated.

**Table 4:** Vaccination Recommendations for CAR T Recipients

Killed/Inactivated Vaccines*	Pre-CAR	>3m	> 6m	>6m	>8m	>10m	>12	>18	Interval Between Vaccinations
Influenza^†^	Flu	Flu							Yearly
RSV^†^		RSV							ACIP guidance
SARS-Cov^†^	SARS-CoV-2	SARS-CoV-2							ACIP guidance for immunocompromised patients
Pneumococus^‡^			PCV20	titers	PCV20	PCV20			1–2 mo
Diphtheria, tetanus, and acellular pertussis (DTap) ^§^,^∥^			DTap	titers	Td	Td			1–2 mo
Hepatitis A ^¶,#^			HAV	titers			HAV		6 mo
Hepatitis B ^#,^**			HAB	titers	HBV		HBV		2 mo
Shingrix^††^							VZV	VZV	

* For inactivated “dead” virus vaccines, vaccination should be at least 2 mo post last dose of IVIG.

† If patient is going to receive CAR T-cell therapy during influenza season, administer annual inactivated influenza vaccine after leukapheresis and ≥ 2 weeks prior to beginning lymphodepletion chemotherapy, (if not previously administered) Subsequent annual vaccinations can resume > 6 mo after CAR T therapy. RSV vaccines guidance by ACIP and ASTCT guidelines

‡ Check titers for S. Pneumonia (IgG, 23 serotypes) 1–2 mo after each PCV20. A positive response to PCV20 is defined: as achieving a seroprotective IgG level against S. pneumoniae in ≥15 out of 20 PCV20 serotypes at 1–2 mo post-vaccination. A positive response requires no further PCV20 vaccination

§ Separate component vaccines (shots) may be used instead for DTaP, IPV, and Hib if Pentacel^®^ is unavailable.

∥ Check titers to Hib, tetanus toxoid.

¶ If NOT administering hepatitis B series using Heplisav-B^®^, Twinrix^®^ can be administered on days when HAV and HBV are given together. (Twinrix^®^ approved for age ≥ 18 y)

# Hepatitis A & B surface antigen IgG.

** Hepatitis B vaccination is accomplished preferably with Heplisav-B^®^ based on data extrapolated from patients with chronic kidney disease or on hemodialysis for ESRF. Alternatively, double (40 mcg/dose = 2 mL total) doses of Engerix-B^®^ may be given. Patients who do not respond to the primary vaccine series should receive an additional 1–3 doses of the same vaccine or, alternatively, repeat series with a different vaccine brand (e.g., double doses of Engerix-B^®^ if did not respond to Heplisav-B^®^ or single doses of Heplisav-B^®^ if did not respond to Engerix-B^®^).

†† Not until ≥ 1 year post CAR T-cell therapy, ≥ 1 year post transplant, ≥ 8 mo off all systemic immunosuppressive therapy for chronic GVHD, and absolute CD4 T cell count > 200 per microliter.

**Table 5:** Post-CAR T Infection Knowledge Gaps and Proposed Future Studies For CAYA And Adults.

Knowledge Gap or Problem	Proposal
Lifetime infection risk is underestimated and not systematically assessed	Long-term longitudinal infection surveillance and monitoring studies to inform future standardized monitoring
The net state of immunosuppression is heterogenous in CAR T recipients	Prospective multicenter studies of immune reconstitution kinetics are needed to inform risk-mitigating strategies, including antibiotic prophylaxis, Ig replacement therapy, and /or vaccine timings.
Distinguishing infections from CAR T toxicities	Development of real-time immune profiling to differentiate CAR T toxicities from infections to inform management
Scant data about infections in survivors with severe prolonged cytopenia and the role of long-term antimicrobial prophylaxis	Prospective randomized studies to determine the optimal antimicrobial prophylaxis and duration.
The efficacy of Ig replacement therapy has been extrapolated from HCT and other non-CAR T settings.	Upcoming randomized control studies may ultimately guide Ig-replacement in the CD19 and BMCA CART contexts.
Antibiotic stewardship linked to microbiome preservation to improve clinical outcomes is still evolving.	Rapid and sensitive diagnostics might become important antibiotic stewardship tools. Multi-institutional collaborative data-sharing might facilitate gathering larger datasets and integrating them for clinical, immunological, and microbiological data analysis to identify trends and risk factors that will advance the field.
Understanding the implications of artificial intelligence (AI) tools	Validation and Integration of AI tools to enhance infection-related patient care outcomes
